# A low-grade chondrosarcoma presenting as an unusual cervical mass in the hyoid bone: a case report

**DOI:** 10.1186/1752-1947-6-21

**Published:** 2012-01-18

**Authors:** Hirohiko Tachino, Hiroaki Fushiki, Masayuki Ishida, Yukio Watanabe

**Affiliations:** 1 Department of Otolaryngology, Head and Neck Surgery, University of Toyama, Toyama, Japan

## Abstract

**Introduction:**

A chondrosarcoma originating from the hyoid bone is very rare. Here, we describe a case of low-grade chondrosarcoma of hyoid origin and discuss its preoperative imaging features, including those on positron emission tomography-computed tomography, and its recurrence rate.

**Case presentation:**

A 42-year-old Japanese man noticed a mass in the right submandibular region of his neck. A hard 3.0 × 2.8 cm tumor was noted on the right side of his hyoid bone. The mass was immobile and moved with deglutition.

**Conclusion:**

Even though radiographic studies, including positron emission tomography-computed tomography, were inconclusive, the cartilaginous tumor was surgically removed *en bloc*, and the tumor was diagnosed based on the results of pathological investigations. Close follow-up is recommended in such cases due to the potential for recurrences, because local recurrence occurred in 50% of the reported cases of grade one chondrosarcomas.

## Introduction

Chondrosarcoma, a malignant tumor characterized by the production of a cartilage matrix, accounts for about 11% cases of primary malignant bone tumors [1]. The parts of the body where it develops at relatively high incidences include the long bones, the pelvis and the ribs [1]. The head and neck area are seldom affected; chondrosarcomas in this area reportedly account for 1% to 12% of all reported cases of the disease [1]. Chondrosarcoma of the hyoid bone is extremely rare, with only 15 cases being reported in international literature. Here, we describe a case of low-grade chondrosarcoma of hyoid origin. We also discuss its preoperative imaging features and its recurrence rate.

## Case presentation

A 42-year-old Japanese man noticed a mass in the right submandibular region of his neck in June 2010. He visited the department of otolaryngology at a nearby general hospital the next month and was referred for medical treatment. More than two weeks later, he visited our University Hospital. A hard 3.0 × 2.8 cm tumor was noted on the right side of his hyoid bone. The mass was immobile and moved with deglutition. No spontaneous or tender pain was noted.

**Figure 1:**
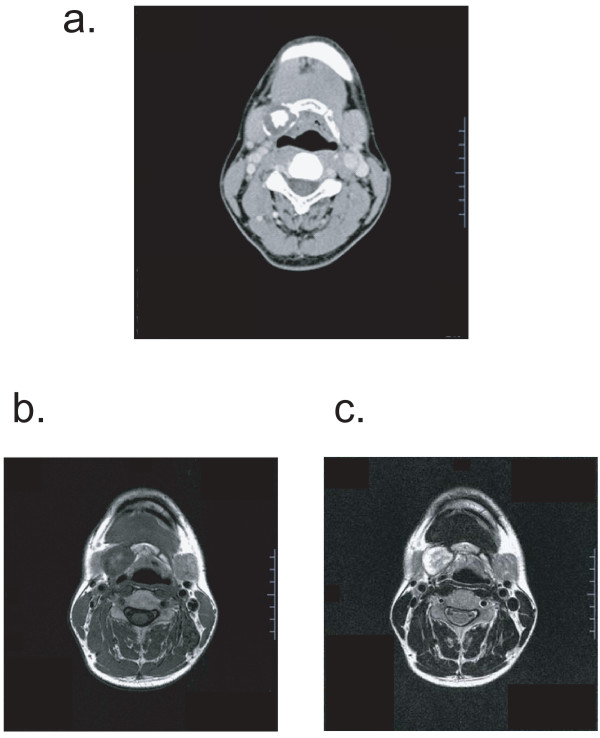
**Preoperative imaging by CT and MRI**. **(a) **Computed tomography of his neck. A gourd-shaped distension of bone was present on the right side of his hyoid bone. A multilocular cystic area of low density was observed in the distention. Central ossification was evident in the anterior lesion. **(b) **Magnetic resonance imaging T1-weighted image with low signal intensity. **(c) **MRI T2-weighted image high signal intensity. A mosaic pattern was visible in the high signal area.

**Figure 2:**
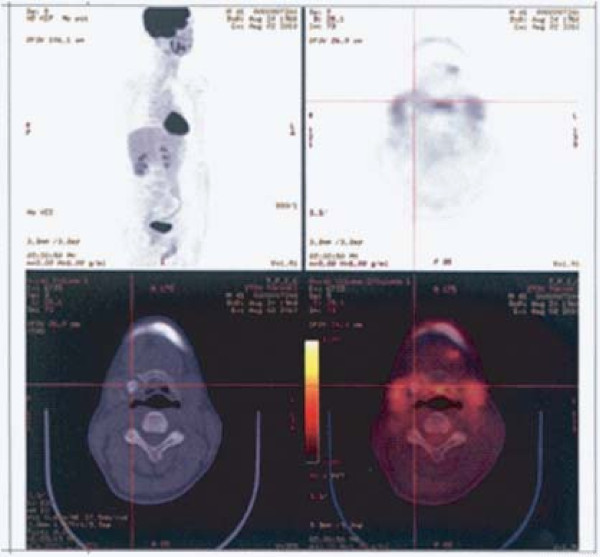
**Positron emission tomography-computed tomography**. No abnormal accumulation in the hyoid tumor or systemic abnormalities were evident.

Laryngeal endoscopy revealed no contributory abnormal findings. Computed tomography (CT) demonstrated a gourd-shaped distension of bone in the area extending from the body of the hyoid bone to the right greater horn. A multilocular cystic area of low density was observed inside the distension, and central ossification was evident in the anterior lesion (Figure 1a). Magnetic resonance imaging (MRI) of his neck revealed a gourd-shaped nodular lesion of 2.5 × 2.2 cm (upper part) and 1.6 × 1.0 cm (lower part) on the right side of his hyoid bone. The signal intensity was low in T1-weighted images and high in T2-weighted images, with a mosaic pattern being observed in the high signals (Figure 1b, c). Based on these findings, a cartilaginous tumor originating from the right side of his hyoid bone was diagnosed. No abnormal accumulation was detected by bone scintigraphy or fluorodeoxyglucose positron emission tomography (FDG-PET) (Figure 2). Therefore, high-grade malignant chondrosarcoma was diagnosed by exclusion. An analysis of his blood samples revealed no contributory abnormalities.

Surgery was performed to extirpate the tumor. His geniohyoid, mylohyoid, sternohyoid, and thyrohyoid muscles were detached from his hyoid bone. The tumor mass and surgical margins were resected bilaterally on the left side of the body of his hyoid bone and the right greater horn. There were almost no adhesions between the tumor and the tissue around it. Following a favorable postoperative course, our patient was discharged from hospital.

**Figure 3:**
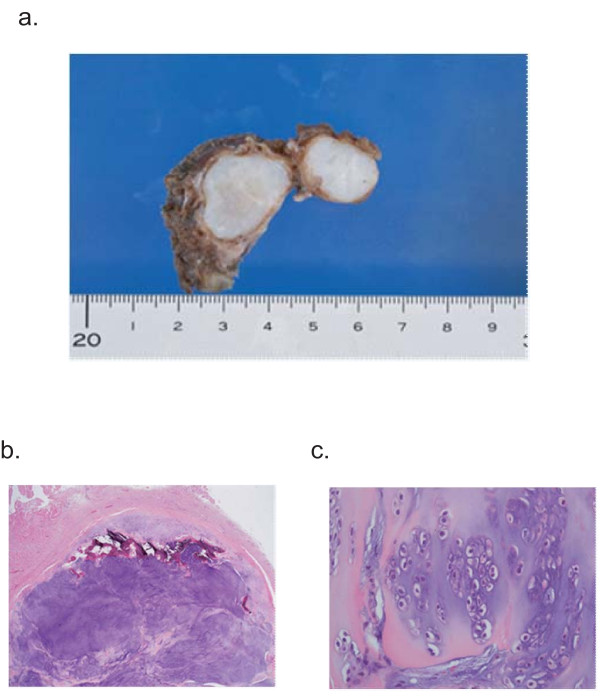
**Macroscopic and histopathological examination**. **(a) **The extirpated specimen was revealed to be a semi-lobular cartilaginous tumor mass covered by a thin fibrous coat. **(b,c) **Histopathological findings: (b) hematoxylin-eosin (HE)-stained, ×2; (c) HE-stained, ×40. The cartilaginous tumor mass is relatively well differentiated, the cell density is somewhat high and many binuclear cells were seen; an open chromatin pattern is evident with intranuclear structures observable in many cells.

A macroscopic examination identified the cartilaginous tumor as a semi-lobular mass covered by a thin fibrous coat. Histopathological examination revealed that it was relatively well differentiated, but with a somewhat high cell density and many binuclear cells. An open chromatin pattern was evident and intranuclear structures were observed in many cells. The condition was diagnosed as chondrosarcoma, grade one (Figure 3a-c). No additional surgical treatment was required. Our patient has been periodically followed up at our outpatient clinic. Twelve months postoperatively, the clinical course has been favorable and no local recurrences or remote metastases have been detected.

## Discussion

Generally, the most useful diagnostic imaging examination for chondrosarcoma is CT, which detects irregular bone destruction. Intratumoral calcification is noted in about 75% of patients using this tool [2,3]. MRI is useful for surgical planning and for evaluating the degree of invasion of the tumor into the surrounding soft tissues. Signal intensity is low in T1-weighted images and high in T2-weighted images; the calcified portion is signal-free. A characteristic finding is a mosaic pattern in high signal areas in T2-weighted images [4]. In our case, a CT examination demonstrated no evidence of bone destruction, but revealed intratumoral calcification. On MRI examination, the signal intensity was low in T1-weighted images and high in T2-weighted images, with a mosaic pattern; however, no invasion into the tissue around the lesion was seen.

In most cases, bone scintigraphy examination reveals poor uptake in the chondrosarcoma itself and morbid uptake in the destroyed area of the affected bone [5]. FDG-PET is useful for grading tumors and for evaluating local recurrences and metastatic diseases [6]. A standardized uptake value of 1.3 has been reported as a border value that distinguishes between benign and malignant tumors [7]. However, bone scintigraphy and FDG-PET did not rule out the presence of chondrosarcoma in our case. The negative test results may have been obtained partly because the hyoid bone itself is small, and partly because the reactive bone formation was inconspicuous on histology. Therefore, it may be impossible to distinguish chondroma from low-grade chondrosarcoma of hyoid origin by means of preoperative imaging tests, including positron emission tomography-computed tomography (PET-CT).

Generally, the occurrence of metastases becomes more probable as the degree of malignancy increases, resulting in poor prognoses [8]. The five- year survival rate for all cases of chondrosarcoma is reportedly 90% for grade one, 81% for grade two and 43% for grade three [9]. However, in the few grade one cases of hyoid origin with available outcome records, local recurrences occurred in five of the 10 reported cases. Therefore, close follow-up is necessary.

## Conclusion

The present case did not involve union of the lesion to the tissue around it. The tumor and surrounding intact hyoid bone tissue were completely resected. Pathological results led to the diagnosis of a grade one tumor of low malignancy. Even though our radiographic studies, including PET-CT, were inconclusive, we surgically removed the cartilaginous tumor *en bloc *and the tumor was diagnosed based on the results of pathological investigations. Chondrosarcomas of hyoid origin have a high incidence of local recurrence and are sometimes metastatic. Therefore, it seems necessary to closely follow-up the patient for a prolonged period of time.

## Consent

Written informed consent was obtained from the patient for publication of this case report and any accompanying images. A copy of the written consent is available for review by the Editor-in-Chief of this journal.

## Competing interests

The authors declare that they have no competing interests.

## Authors' contributions

HT was involved in drafting the manuscript and reviewing the literature. HF was a major contributor in revising the manuscript and getting informed consent from our patient. MI was involved in reviewing the literature. YW reviewed the manuscript. All authors were responsible for the diagnosis, treatment and follow-up of the patient described in this case report. All authors read and approved the final manuscript.
